# Psychometric validation of the Columbia-Suicide Severity rating scale in Spanish-speaking adolescents

**DOI:** 10.25100/cm.v43i4.2294

**Published:** 2017-12-30

**Authors:** Daniel Serrani Azcurra

**Affiliations:** 1 Neuropsychology and Development Psychology Program from the Department of Interdisciplinary Studies, Biology, Psychology and Culture. Faculty of Psychology. Universidad Nacional de Rosario. Rosario, Argentina.

**Keywords:** Columbia-Suicide Severity Rating Scale, adolescents, psychometric validation, cut-off scores, Escala de Columbia de severidad de suicidio, adolescentes, validación psicométrica, puntos de corte

## Abstract

**Introduction:**

Adolescent suicide is a major public health issue, and early and accurate detection is of great concern. There are many reliable instruments for this purpose, such as the Columbia-Suicide severity rating scale (C-SSRS), but no validation exists for Spanish speaking Latin American adolescents.

**Objetive:**

To assess psychometric properties and cut-off scores of the C-SSRS in Spanish speaking adolescents.

**Methods:**

Exploratory assessment with principal component analysis (PCA) and Varimax rotation, and confirmatory analysis (CFA) were performed on two groups with 782 and 834 participants respectively (N=1616). Mean age was 24.8 years. A Receiver operator analysis was applied to distinguish between control and suicide-risk subgroups adolescents.

**Results:**

Promax rotation yielded two 10-items factors, for suicide ideation and behavior respectively. C-SSRS was positively correlated with other suicide risk scales, such as Beck Depression Inventory-II, Suicidal Behaviors Questionnaire-Revised, or PHQ-9. Confirmatory factor analysis yielded a two-factor solution as the best goodness of fit model. C-SSRS showed adequate ability to detect suicide risk group with positive predictive value of 68.3%. ROC analyses showed cutoff scores of ≥ 6 and ≥ 4 for suicide ideation and behavior scales respectively

**Conclusion:**

This research offers data supporting psychometric validity and reliability of C-SSRS in nonclinical Spanish-speaking students. Added benefits are flexible scoring and management easiness. This questionnaire yields data on distinct aspects of suicidality, being more parsimonious than separate administration of a bunch of questionnaires.

## Resumen

**Introducción:**

Suicidio adolescente es un problema de salud pública y su detección temprana es de gran interés. Hay numerosos instrumentos confiables para este objetivo, como la escala Columbia para la detección del riesgo de suicidio, pero no ha sido validada para población adolescente de habla hispana en Latinoamérica.

**Objetivo:**

Validar psicométricamente y explorar los puntos de corte para la escala Columbia en adolescentes de habla hispana.

Métodos: Analisis exploratorio con extracción de componentes principales y rotación Varimax así como análisis confirmatorio fueron llevados a cabo sobre 782 y 834 participantes respectivamente (N=1616). La edad media fue de 24.8 años. El análisis ROC distinguió entre controles y adolescentes en riesgo de suicidio.

**Resultados:**

La rotación Promax arrojó dos factores de 10 ítems, para ideación y comportamiento suicida respectivamente. La C-SSRS correlaciono positivamente con otras escalas de detección de riesgo de suicidio como Beck Depression Inventory-II, Suicidal Behaviors Questionnaire-Revised, o el PHQ-9. El análisis factorial confirmatorio ofreció una solución de 2 factores como el modelo con mejor ajuste. El análisis ROC tuvo puntos de corte ≥ 6 y ≥ 4 para las escalas de ideación y comportamiento suicida respectivamente

**Conclusión:**

Esta investigación ofrece datos que apoyan la validez psicométrica y confiabilidad de la C-SSRS en población de estudiantes adolescentes hispano-hablantes latinoamericanos. Beneficios adicionales son un sistema de puntaje flexible y facilidad de administración. Este cuestionario ofrece datos sobre distintos aspectos de suicidalidad siendo más robusto que la administración separada de varios cuestionarios diferentes.

## Introduction 

Suicide is one of the most important and yet unresolved public health problem. Suicide rates have been growing worldwide with an estimate of over 800,000 deaths and almost ten suicide attempts for each death. According to the World Health Organization, every 40 seconds, a person commits suicide being the second leading cause of death among 15-29 year old ^1^ ^,^ ^2^. High suicidality rates are a widespread concern, particularly in Western ^3^ and Central Europe ^4^, the U.S. ^5^, Asiatic countries such as Russia and former Socialist Republics ^6^, South Korea ^6^, China ^7^, India ^8^, Sri Lanka ^9^; and Latin American countries such as Cuba ^10^, Uruguay ^11^; Ecuador ^12^, Bolivia ^13^, Brazil ^14^, Argentina ^15^ and Colombia ^16^. Suicide risk increases in people with mental disorders or impulsive behavior, those facing stressful situations or with easy access to harmful tools such as poisoning, hanging and firearms ^17^. Besides that, suicide is three times more common in men than women ^18^. Suicide is a prominent risk in adolescents. In a recent ecological study on suicide mortality including 19 American countries from 2001 to 2008, the mean suicide rate for people between 10-24 years was 5.7/100,000 (males: 7.7/100,000, females: 2.4/100,000) and estimated lifetime prevalence of suicide ideation, plans, and attempts between 13-18 years were 12.1%, 4.0%, and 4.1%, respectively. Fear, distress and substance abuse were most significant predictors ^19^. Adolescent suicide rates are rising in Argentina (7.9/100,000), and decreasing in Canada and Colombia ^20^. Suicidal ideation is a risk factor for attempts and completed suicide and the same is true about non-suicidal self-injuries ^21^. Despite the importance for accurate assessment of suicide risk between young people, very few instruments have proved to be reliable enough to this purpose due to low validity or negative predictive likelihood ^22^. The Columbia Suicide Severity Rating Scale (C-SSRS) is available for free at www.cssrs.columbia.edu and has been widely used for assessment of suicidality by several agencies such as the US Substance Abuse and Mental Health Services Administration's Center for Integrated Health Solutions, the National Institute of Mental Health, the US Food and Drug Administration, US National Library of Medicine, World Health Organization (WHO), American Medical Association (AMA) Best Practices, Health Canada, Korean Association for Suicide Prevention, Japanese National Institute of Mental Health and Neurology and the Israeli Defense Force ^23^, The C-SSRS was developed as part of the Treatment of Adolescent Suicide Attempters (TASA) study, which assesses suicide risk in different clinical and trial settings, from inpatient psychiatric facilities to outpatient primary care and emergency departments. This scale assesses worst point and lifetime severity and intensity of suicidal ideation, and type and lethality of suicidal behavior. Selected items are strong predictors of suicide risk, including preparatory activity ^24^. This scale has been translated into 103 languages, including Spanish. Psychometric properties of the C-SSRS were evaluated in three multisite, double blind studies with adolescents showing high internal reliability (α= 0.73 to 0.95) and good convergent validity (r= 0.80) with well-known suicidal instruments ^25^. Compared with the Columbia Suicide History Form, the C-SSRS had high specificity and sensitivity in correctly identifying lifetime and actual aborted and interrupted suicide attempts. A computer automated version of the C-SSRS using interactive voice response technology (e-C-SSRSTM) demonstrated high predictive ability and moderate sensitivity and specificity rates ^26^. In another study ^27^ the e-C-SSRSTM had better sensitivity and specificity than the Item-9 of Patient Health Questionnaire (PHQ-9) for predicting suicide (95.0% and 95.0% vs. 92.0% and 81.0% respectively). A strong interrater reliability of the C-SSRS for discriminating suicidal from non-suicidal behaviors, and detecting five different suicidal behaviors categories (Kappa= 0.90 and 0.88, respectively) was found in a prospective research of delinquent adolescent girls followed up to early adulthood. The C-SSRS subscale of suicide ideation intensity predicted both return to emergency department and future suicide attempts ^27^. During an exploratory study ^28^ to examine concurrent validity of the Scale for Suicidal Thinking-Plus, the Sheehan-Suicidality Tracking Scale and the C-SSRS to detect self-harm and suicidal ideation and behavior, the three scales showed acceptable agreement in detecting passive and active ideation; completed suicide; preparatory actions; and self-injurious behavior, but only the C-SSRS was able to further detect combined categories or aborted and interrupted attempt. Spanish- translated version of the C-SSRS hasn´t been validated for Latin American Spanish-speaking population, and considering that rating scales should be validated respecting the population's cultural and linguistic values for which they are used, this study was designed to assess the psychometric properties of the C-SSRS Spanish version in a Latin-American high-school students sample. The specific objectives were: a) evaluate internal consistency, criterion validity and reliability estimates of the C-SSRS using Confirmatory Factor Analysis, b) set cutoff scores for the scale using Receiver Operating Characteristic (ROC) analyses, and c) examination of sensitivity, specificity, positive and negative predictive values for C-SSRS.

## Materials and Methods

### Participants

Subjects were randomly recruited from a public university in Argentina. Sample size was calculated according to the following formula:

$$n=\frac{t2Xp\left(1-p\right)}{m2}$$

In which *n* is the required sample size, *t* is the confidence level at 99%, *p* is the estimated prevalence of suicide in the area, and *m* is the margin of error at 5%. Were included students which assisted at university courses between 1^st^ March 2015 and 1^st^ May 2015, aged between 18 and 35 years old, and exploratory and confirmatory psychometric analysis were performed with two randomly selected groups with 782 and 834 participants respectively (N= 1,616). Submissions of 78 respondents from both groups were discarded due to missing data. Final sample included group 1 with 737 participants, 442 women (60%) and 295 men (40%) and group 2 with 801 participants, 489 women (61%) and 312 men (39%). Mean age of participants was 25.6 ±7.3. No statistical differences were found between both groups on age (boys 23.13± 5.43 vs. girls 24.15 ±7.01; *t*(988)= 1.87, *p*= 0.80, ns). Written informed consent was obtained from all respondents. Study protocol was approved by the Faculty Ethics Committee. Face-to-face interviews were carried out by senior research interviewers trained in questionnaire administration procedures, and participants completed the questionnaire package in small groups of 15 to 20 during the same regular school day. 

### Instruments

*The 12-item Short Form Health Survey* ^29^ is a 12-item self-report measure of health-related quality of life assessing eight dimensions: physical functioning, role limitation-physical, bodily pain, general health, vitality, social functioning, role limitation-emotional, and mental health, which provides scores for physical and emotional health (higher scores have poorer quality of life).

*Brief version of the Social Support Questionnaire* ^30^ which uses scores for satisfaction with support ranging from 1 (very satisfied) to 6 (very dissatisfied); a cut-off point of ≥2 indicated dissatisfaction with social support.

*Alcohol Use Disorders Identification Test (AUDIT* ^31^ has been validated for use in the community; we used a cut-off ≥8 to identify hazardous drinking; *Drug use* Questions asked if participant ever used or misused street drugs prescribed for medical reasons, rated as yes (1) or no (0).

*Demographic data* Included student self-reported gender and grade level, and socioeconomic status (SES), which was coded as a binary variable with 1 (above) and 0 (below) the minimal national wage.

*The Scale for Suicide Ideation (SSI)* ^32^ is a clinician-rated, semi-structured interview scale consisting of 19 items that evaluate active and passive suicidal desire and specific plans. Each item is rated on a 3-point scale with a cut-off point of ≥6. Higher scores are associated with greater severity of suicide ideation.

*The Beck Depression Inventory-II (BDI-II)* ^33^ ^)^ is a 21-item self-report instrument to assess depression severity. Items are rated from 0 to 3 and scores range from 0-9 (minimal), 10-16 (mild), 17-29 (moderate), and 30-63 (severe). The BDI-II has adequate internal consistency (0.93). 

*Suicidal Behaviors Questionnaire-Revised (SBQ-R)* ^34^ ^)^ evaluates past suicide ideation, frequency, threats, attempts and self-reported suicide likelihood. It has a Cronbach´s α of 0.87, and a cut-off point of ≥7 yields sensitivity between 83.0%-92.5%, and specificity between 90.6%-96.0%; in adolescent psychiatric inpatients a score of 8 has sensitivity of 80% and specificity of 91%. 

*Beck Hopelessness Scale (BHS)* ^35^ is a true-false self-report instrument composed of 21 items measuring hopelessness about future events. It has good psychometric properties. Severity of hopelessness is as follows: 0-3 minimal, 4-8 mild, 9-14 moderate, and 15-20 severe. Hopelessness has been associated with suicide ideation and attempts in adolescents and young adults ^36^.

*Satisfaction with Life Scale (SWLS)* ^37^ ^)^ is a 5-item, self-report questionnaire that assess satisfaction with life. Items are rated on a 7-point Likert scale, has a Cronbach's α of 0.87 and adequate temporal validity (*r*_tt_ = 0.54) at four years. 

*The Positive and Negative Affect Schedule (PANAS)* ^38^ is a 20-item self-report instrument to assess positive (PANAS-PA; 10 items) and negative (PANAS-NA; 10 items) affects. Items are rated on a 5-point Likert scale, and has adequate reliability and validity for PANAS-PA and PANAS-NA scales (α = 0.88 and 0.84 respectively).

*PHQ-9* ^39^ is a self-administered instrument to detect major depression and is focused on preceding 14 days and asks how often the participant has been bothered by something or someone. Items are scored on a 0-3 scale, and include little pleasure, feeling down, sleep disturbance, fatigue, appetitive disturbances, feelings of failure or guilt, concentration difficulty, psychomotor retardation or agitation, and suicidal ideas. Scores of 5, 10, 15, and 20 correspond to mild, moderate, moderately severe, and severe levels of depression.

*The Schedule for Affective Disorders and Schizophrenia for School-Aged Children-Present and Lifetime (KSADS-PL)* ^40^ is a semi-structured diagnostic interview. Suicidal behavior is determined with four items: item-1 suicidal thoughts, item-2 and 3 suicide attempts seriousness and lethality and item-4 self-harming behavior without intent to die. It is considered a reliable instrument for adolescent population concerning suicidality with inter-rater reliability weighted kappa of 0.87.

*The Columbia Suicide Severity Rating Scale (C-SSRS)* was developed by researchers from Columbia, Pennsylvania and Pittsburgh Universities to evaluate suicidal ideation and behavior and has been translated into a myriad of languages including Spanish. It is composed of four categories: severity and intensity of suicidal ideation, suicidal behavior and lethality. The scale uses a lifetime and worst point timeframe. The Lifetime/Recent version records past and recent suicidality, Last Visit version assesses suicidality since patient's last visit. Screener version is used in ER settings and crisis call centers for non-mental health users. In the present study the C-SSRS categories were rearranged to facilitate definitions and improve outcomes report (Table 1). *Suicidal Ideation* has 6 mutually exclusive items scored in an ordinal scale (total score: 0-30): severity (0-5), frequency (0-5), duration (0-5), controllability (0-5), deterrents (0-5), and reasons (0-5). *Suicidal Behavior* has 2 mutually exclusive items scored in an ordinal scale (total score: 0-10): intensity (0-5) and lethality (0-5). In case that any participant endorsed active suicidal ideation with plan or intent to act on it, was prompted to arrange further evaluation with a mental health team or to the emergency room, to ensure proper management of the event. 

**Table 1:** C-SSRS categories and sub-items

Category	Item
I: Severity of Suicidal Intention	1: Wish to be dead
	2: Non-specific Active Suicidal Thoughts
	3: Active Suicidal Ideation with any methods (not plan) without intent to act
	4: Active Suicidal Ideation with some intent to act, without specific plan
	5: Active Suicidal Ideation with specific plan and intent
II: Intensity of Suicidal Ideation	6: frequency (from 1: < once a week to 5: many times each day)
	7: duration (from 1: Fleeting to 5: More than 8 hours/persistent or continuous)
	8: controllability (from 1: Easily control thoughts to 5: Unable to control)
	9: deterrents (from 1: stopped from suicide to 5: definitely did not stop suicide)
	10: reasons (from1: get attention, revenge, reaction from others to 5: end or stop the pain (can´t go on living with pain or how you feel)
III: Suicidal Behavior	11: Preparatory Acts or Behavior
	12: Aborted Attempt
	13: Interrupted Attempt
	14: Actual Attempt (non-fatal)
	15: Self-injurious behavior without suicidal intent
IV: Lethality	16: No or very minor physical damage
	17: Minor physical damage
	18: Moderate physical damage; medical attention needed.
	19: Moderately severe physical damage; *medical* hospitalization and likely intensive care required.
	20: Severe physical damage; *medical* hospitalization with intensive care required
	21: Death

### Procedure

Participants completed questionnaires assessing distressful states, quality of life, drugs or alcohol consumption and social support. After that they were administered the clinical instruments assessing depressive mood, suicide risk, quality of life and finally the C-SSRS. The sample was divided into suicidal and non-suicidal subgroups to assess the C-SSRS discriminant and criterion-related validity 

Inclusion criteria for the suicidal subgroup included:

1. SBQ-R total score of ≥7 ^41^
2. BDI-II total score of ≥10 (mild -to-severe depressive symptoms) ^42^
3. K-SADSPL score of 2 on item-1 (mild suicidal ideation) and a score of 2 on any of items 2-4 (mild suicidal acts) regardless of ideation ^43^, and
4. PHQ-9 score ≥8 ^44^

Using these criteria, 138 participants (9%), 82 women and 56 men were assigned to the suicidal subgroup, and the remaining 1,400 participants (91%) to the non-suicidal control subgroup. No differences were found between both groups for age (*M* = 23.8 ±5.38) and gender. 

### Statistical analysis 

Quantitative variables were reported by mean and standard deviation (±SD) for comparing variables; otherwise Student's t-test was used. Categorical variables were evaluated by χ^2^ test or Fisher's exact test. Group 1 was used to perform exploratory factor analysis. Internal consistency was determined with Cronbach's alpha coefficient. Floor and ceiling effects were calculated not exceeding the limit of 15% of participants. Items correlation was assessed with Bivariate Spearman rank coefficient and effect sizes were computed with Cohen's d. Factorial structure and construct validity of the C-SSRS was assessed with principal component analysis (PCA) and Promax rotation. Kaiser-Meyer-Olkin and Bartlett's sphericity tests were used to determine data adequacy for factorial processing. 

Scale factors were elicited using Velicer's minimum average partial correlation (MAP) and Cattell's scree tests. Confirmatory factor analysis (CFA) was conducted with group 2 (N= 801), using EQS software ^44^. Data was treated as continuous using a zero-order correlation between factors (default model) and 1, 2 and 4 factor structure (restrictive models). Item's loadings and factor correlations in the first (default) model were set to 0 and released to vary at freedom degrees of adjustment, while confined to adjust to 1, 2 or 4 factors in the restrictive model. A corrected Satorra-Bentler X^2^ was used to allow for non-normality and robust goodness-of-fit indices. Criterion for goodness-of-fit was set at 0.90 for Incremental fit index (IFI) ^45^, Comparative fit index (CFI), Goodness-of-Fit Index (GFI), Adjusted Goodness-of-Fit Index (AGFI) and normative fitness index (NFI); 0.80 for Root mean square error of approximation (RMSEA) ^46^; 0.7 for non-normative fitness index (NNFI) and Parsimonious norm fit index (PNFI) and a 2:1 or 3:1 range for Chi-square minimal simple discrepancy divided by freedom degrees (CMINDF). Concurrent validity was examined with SSI, BDI-II, SBQ-R, BHS, SWLS, PANAS, and KSADS-PL using Pearson Correlation. Criterion validity was assessed applying *t* test and one-way MANOVA between suicidal and non-suicidal subgroups. Relative contribution of sociodemographic and psychiatric factors, together with C-SSRS subscales to suicidal risk was assessed using stepwise multiple regression analysis ^47^. Receiver operator characteristics (ROC) analysis was performed to evaluate screening properties of C-SSRS, cutoff threshold was defined by optimal trade-off between sensitivity and specificity (Youden's index) ^48^. For all the tests, the accepted significance level was 0.01 %. Analyses were carried out using SPSS 15.0 (SPSS, Chicago, IL, USA) 

## Results 

Table 2 describes sociodemographic data and ratings on the C-SSRS. Pooling together groups 1 and 2 (N= 1,538) the mean C-SSRS score was 1.21 ±0.76. Females had higher scores than males (1.29 ±0.45 vs. 1.11 ±0.65, *t* = 0.765, *p* <0.09) although this difference was not statistically significative. 

**Table 2:** Participant's demographic data (including groups 1 and 2)

Variable	Male (n = 607)	Female (n = 931)	*p* <0.01
Age (Range)	18-35	18-35	ns
Age (mean ± SD)	23.13±5.43	24.15±7.01	ns
Education (Range)	13-18	13-20	ns
Education (mean ± SD)	15.80 ± 2.2	17.1±3.5	0.31
AUDIT	0.59±0.11	0.45±0.19	0.02
12-item Short Form measure of quality of life	3.34±1.37	3.46±1.16	0.51
Brief version of the Social Support Questionnaire	1.24±0.12	1.32±0.30	0.34
C-SSRS total score (mean ± SD)	1.14±0.35	1.38±0.22	0.05
Suicide severity score (mean ± SD)	0.12±0.13	0.18±0.12	0.14
Suicide behavior score (mean ± SD)	0.22±0.07	0.20±0.12	0.01
Suicide intensity score (mean ± SD)	1.21±0.16	2.18±0.17	0.04
Suicide lethality score (mean ± SD)	1.18±0.16	1.09±0.12	0.01
PHQ-9	5.21±1.11	5.98±1.99	0.56
SSI	2.21±1.17	2.12±1.19	0.39
BDI-II	7.19±2.11	8.65±2.40	0.62
SBQ-R	3.19±1.09	4.22±1.43	0.82
BHS	3.17±1.18	3.00±1.64	0.23
SWLS	20.99±6.10	25.01±7.31	0.43
PANAS-PA	28.11±12.10	33.46±10.19	0.29
PANAS-NA	17.19±8.12	18.93±8.55	0.29
KSADS-PL item 1	1.06±0.22	1.04±0.19	0.29
KSADS-PL item 2	1.08±0.21	1.10±0.32	0.22
KSADS-PL item 3	1.14±0.09	1.10±0.12	0.33

SSI: Scale for Suicide Ideation; BDI-II: Beck Depression Inventory; SBQ-R: Suicidal Behaviors Questionnaire-Revised; Beck Hopelessness Scale (BHS); SWLS: Satisfaction with Life Scale; PANAS-PA: Positive Affect Schedule; PANAS-NA: Negative Affect Schedule; KSADS-PL: Schedule for Affective Disorders and Schizophrenia for School-Aged Children-Present and Lifetime

### Internal consistency 

The C-SSRS ideation subscale yielded a Guttmann split-half reliability of .91 and a Cronbach's alpha of 0.87, 0.89 and 0.93 for the whole sample, the suicide-risk and control groups respectively, with good internal consistency. Suicide intensity, severity and behavior subscales had Cronbach's alpha of 0.73, 0.89 and 0.91 respectively. Lowest scores for floor and ceiling effects were found in 11.8%, 10.5%, 10.1%, and 11.3% of cases in severity, behavior, intensity and lethality subscales respectively. Highest scores were for intensity subscale (2.4%). C-SSRS inter-items mean correlation was 0.64 (minimum= 0.47, maximum= 0.72). Item-total C-SSRS corrected correlation score are presented in Table 3, with values ranging from 0.47 for item 6 and 0.74 for item 5 (*p* <0.001).

**Table 3:** C-SSRS Internal Consistency and Mantel-Haenszel Statistic (αMH)

Item	M	SD	r_tot_	α	α_MH_
Severity of Suicidal Intention					
1. wish to be dead	0.15	0.01	0.62	0.926	0.68
2. Non-specific Active Suicidal Thoughts	0.12	0.02	0.49	0.929	1.04*
3. Active Suicidal Ideation with any methods (not plan) without intent to act	0.13	0.04	0.54	0.926	1.30
4. Active Suicidal Ideation with some intent to act, without specific plan	0.11	0.03	0.56	0.926	1.44
5. Active Suicidal Ideation with specific plan and intent	0.14	0.01	0.74	0.926	0.89
Intensity of Suicidal Ideation					
6. frequency (from 1: < once a week to 5: many times each day)	1.35	0.73	0.47	0.925	2.41
7. duration (from 1: Fleeting to 5: More than 8 hours/persistent or continuous)	1.56	0.79	0.67	0.925	1.31
8. controllability (from 1: Easily control thoughts to 5: Unable to control)	2.43	0.98	0.55	0.923	0.99*
9. deterrents (from 1: stopped from suicide to 5: definitely did not stop suicide)	2.05	0.87	0.54	0.924	0.89
10. reasons (from1: get attention from others to 5: end or stop the pain	1.26	0.65	0.72	0.925	1.14
Suicidal behavior					
11. Preparatory Acts or Behavior	0.22	0.17	0.65	0.925	1.11
12. Aborted Attempt	0.17	0.05	0.63	0.925	0.84
13. Interrupted Attempt	0.24	0.10	0.48	0.924	1.34
14. Actual Attempt (non-fatal)	0.15	0.09	0.50	0.926	1.48
15. Self-injurious behavior without suicidal intent	0.21	0.19	0.61	0.925	1.33
16. No or very minor physical damage	0.12	0.07	0.53	0.928	1.18
Suicide Lethality					
17. Minor physical damage	1.80	0.39	0.54	0.925	1.95
18. Moderate physical damage; medical attention needed	0.84	0.46	0.61	0.928	1.53
19. Moderately severe physical damage; *medical* hospitalization	1.01	0.32	0.64	0.932	1.49
20. Severe physical damage; *medical* hospitalization with intensive care	1.03	0.27	0.57	0.930	1.37

* Statistically significant at *p* <0.01.

### Factor validity

#### Exploratory factor analysis

A principal axis analysis with Promax rotation was conducted for the correlation matrix of the C-SSRS, as both Kaiser-Meyer-Olkin test of sample adequacy (KMO = 0.92) and Bartlett's sphericity test (χ^2^ = 2,740.4, *p* <0.001) indicated that factor model was adequate for data processing. Velicer's minimum average partial correlation test and scree plot yielded a two-factor solution. All items had factor loadings ≥0.40 (Table 4).

**Table 4:** C-SSRS Factor loadings

Item	pattern matrix		structure matrix		Commonalities	
	1**^st^** Factor	2**^nd^** Factor	1**^st^** Factor	2**^nd^** Factor	initial	extraction
1.Wish to be dead	0.83	0.19	0.85	0.25	1.00	0.80
2. active Suicidal Thoughts Non-specific	0.82	0.14	0.82	0.18	1.00	0.79
3. Active Suicidal thoughts any methods not plan no intent to act	0.80	0.17	0.78	0.21	1.00	0.78
4. Active Suicidal thoughts some intent to act no specific plan	0.79	0.29	0.77	0.25	1.00	0.76
5. Active Suicidal thoughts specific plan and intent	0.76	0.23	0.74	0.26	1.00	0.73
6. frequency (1: < once/week - 5: many times each day)	0.75	0.13	0.73	0.20	1.00	0.72
7. duration (1: Fleeting -5: >8 hours/persistent or continuous)	0.74	0.18	0.72	0.28	1.00	0.71
8. controllability (1: Easily control thoughts-5: Unable to control)	0.73	0.17	0.70	0.27	1.00	0.69
9. deterrents (1: stopped from suicide-5: not stop suicide)	0.71	0.11	0.68	0.20	1.00	0.67
10. reasons (1: get attention from others-5: end or stop pain)	0.70	0.10	0.65	0.19	1.00	0.64
11. Preparatory Acts or Behavior	−0.23	0.86	0.18	0.82	1.00	0.79
12. Aborted Attempt	−0.27	0.82	0.09	0.81	1.00	0.76
13. Interrupted Attempt	−0.30	0.80	0.23	0.78	1.00	0.72
14. Actual Attempt (non-fatal)	−0.19	0.78	0.35	0.77	1.00	0.69
15. Self-injurious behavior without suicidal intent	0.36	0.77	0.08	0.76	1.00	0.68
16. No or very minor physical damage	0.18	0.76	0.19	0.74	1.00	0.68
17. Minor physical damage	0.14	0.74	0.15	0.73	1.00	0.71
18. Moderate physical damage; medical attention needed	0.28	0.69	0.20	0.70	1.00	0.70
19. Moderately severe physical damage; medical hospitalization	0.15	0.68	0.13	0.63	1.00	0.69
20. Severe physical damage; hospitalization intensive care	−0.04	0.67	-0.21	0.68	1.00	0.65

Note Significant factor loading in bold numbers.

The two extracted oblique factors had eigenvalues of 6.97 (95% CI: 5.11-7.75) and 4.91 (95% CI: 3.76-4.61) respectively. First factor contained 10 items (1 to 10) consistent with concept of suicide ideation. Second factor included 10 items (11 to 20) reflecting suicide behavior. Both factors were moderately and positively correlated with each other (r = 0.67). 

### Concurrent validity

One-way multivariate analysis of variance (MANOVA) with the two C-SSRS subscales weighted scores as dependent variables and gender as independent variable showed significant gender differences, women had higher scores in suicide ideation (Hoteling's T2= 0.03; Exact *F*(4, 630) = 4.67, *p* <0.01, d= 0.79) and lower in suicide behavior (*F*(4, 630)= 3.86, *p* <0.05, *d*= 0.82), which is a usual finding in general population. C-SSRS had a positive relationship with SSI (r= 0.71, d= 0.87, *p* <0.001), BDI-II (r= 0.77, d= 0.84, *p* <0.002), SBQ-R (r= 0.47, d= 0.40, *p* <0.099), BHS (r= 0.59, d= 0.47, *p* <0.017), PANAS-NA (r= 0.70, d= 0.88, *p* <0.002), KSADS-PL item 1 (r= 0.87, d= 0.88, *p* <0.001) and negative correlations with SWLS (r= -0.58, d= 0.12, *p* <0.099) and PANAS-PA (r= -0.63, d= 0.20, *p* <0.045) (Table 5).

**Table 5:** Concurrent correlations of the C-SSRS with other scales

	Suicidal ideation			Suicidal Behavior		
	r	d	*p*	r	d	*p*
PHQ-9 items						
little interest or pleasure	0.45	0.75	0.045	0.32	0.46	0.076
feeling down or depressed	0.55	0.81	0.071	0.41	0.45	0.071
sleep disturbance	0.32	0.52	0.043	0.49	0.50	0.055
Fatigue	0.41	0.60	0.042	0.32	0.56	0.063
appetitive disturbances	0.33	0.45	0.077	0.33	0.48	0.067
feelings of failure/guilt	0.51	0.61	0.049	0.43	0.62	0.073
concentration difficulty	0.22	0.60	0.071	0.39	0.70	0.075
psychomotor retardation or agitation	0.31	0.51	0.049	0.48	0.69	0.083
suicidal or self-destructive ideas	0.89	0.90	0.003	0.52	0.80	0.009
SSI	0.71	0.87	0.001	0.43	0.32	0.087
active suicidal desire	0.70	0.85	0.002	0.40	0.23	0.032
specific plans	0.71	0.82	0.001	0.41	0.31	0.063
passive suicidal desire	0.56	0.43	0.087	0.21	0.23	0.066
BDI-II	0.77	0.84	0.004	0.49	0.22	0.032
SBQ-R	0.47	0.40	0.099	0.79	0.81	0.009
BHS	0.59	0.47	0.017	0.50	0.54	0.018
SWLS	-0.58	0.12	0.099	0.11	0.32	0.034
PANAS PA	-0.63	0.20	0.045	0.13	0.32	0.078
PANAS NA	0.70	0.88	0.002	0.66	0.79	0.004
K-SADS-PL items						
Item 1 suicidal thoughts	0.87	0.88	0.001	0.31	0.43	0.012
Item 2 attempts seriousness	0.31	0.44	0.032	0.78	0.87	0.005
Item 3 attempts lethality	0.12	0.53	0.049	0.50	0.77	0.015
Item 4 Self-harming behavior	0.27	0.22	0.067	0.32	0.41	0.070

Bivariate Spearman rank correlation coefficient; SSI: The Scale for Suicide Ideation, BDI-II: Beck Depression Inventory-II, SBQ-R: Suicidal Behaviors Questionnaire-Revised; BHS: Beck Hopelessness Scale, SWLS: Satisfaction with Life Scale, PANAS: Positive and Negative Affect Schedule, KSADS-PL: Schedule for Affective Disorders and Schizophrenia for School; Cohen´s d: <0.3 (small effect), 0.5 (medium effect), >0.8 (large effect)

### Criterion validity

A *t* test between suicidal and non-suicidal subgroup showed significant differences (1.98 ±0.67 vs. 0.31 ±0.12, respectively; *t*(760) = -10.21, *p* <0.001, *d*= 4.56). A stepwise multiple regression analysis was used to assess incremental validity of C-SSRS subscales as independent variables in predicting suicide risk (Table 6). Sociodemographic factors were introduced in the first step, followed by clinical scales and C-SSRS subscales. Suicide ideation and behavior subscales contributed a significant amount to suicide risk prediction (coefficient= 0.58, t= 4.34, *p* <0.001; OR= 6.21, 95% CI= 5.62-7.87 and coefficient= 0.61, t= 5.12, p <0.001, OR= 6.22, 95% CI= 6.00-7.91, respectively). Adjusted R^2^ increased (R^2^= 0.87, F(9,880)= 1,564.31, d= 6.76 *p* <0.001) after C-SSRS subscales were entered in the equation. 

**Table 6:** Logistic regression analysis of C-SSRS subscales predicting suicide risk

Variable	β	SE	Wald	OR (95% CI)	χ^2^	*^_R2_^*
constant	-0.01	-0.11	0.14	0.91 (0.86-1.00)	0.36	0.12
*Step 1*						
gender (0=female)	0.01	-0.12	0.25	1.03 (0.89-1.18)	0.65	0.34
Education	0.07	0.31	0.45	1.06 (0.59-1.28	0.55	0.43
SES	0.21	0.21	0.39	1.12 (0.87-1.21	0.31	0.54
*Step 2*						
AUDIT	0.06	0.22	0.36	2.21 (1.69-2.52	0.43	0.49
QOL 12-item	0.35	0.48	0.17	1.98 (0.77-1.88)	032	0.31
SSQ	0.37	0.20	0.15	2.11 (1.32-2.68)	0.21	0.48
*Step 3*						
C-SSRS subscales	1.99	0.65	5.98	6.87 (4.87-8.43)	6.87***	0.87
suicide ideation	1.67	0.78	6.31	6.21 (5.62-7.87)	7.12***	0.76
suicide Behavior	1.87	0.55	7.31	6.22 (6.00-7.91)	8.32**	0.82

** p <0.01; *** p <0.001

### Confirmatory factor analysis 

The result of the CFA with the default and restricted 1, 2 and 4 factors models, together with factor correlation scores are shown in Tables 7 and ^8^. The two-factor model elicited the best goodness-of-fit.

**Table 7:** Goodness-of-fit statistics

	X^2^	*df*	significance	CMINDF	IFI	RMSEA	CFI	GFI	AGFI	NFI	NNFI	PNFI
Default model (2 factor)	0.455	566	0.23	4.091	0.817	0.142	0.821	0.623	0.531	0.487	0.518	0.491
Restricted models												
1 factor	0.497	566	0.65	1.098	0.946	0.067	0.947	0.910	0.901	0.899	0.893	0.890
2 factors	0.288	566	0.01	0.872	0.976	0.038	0.980	0.998	0.994	0.997	0.992	0.995
4 factors	0.528	566	0.09	5.673	0.821	0.099	0.812	0.341	0.369	0.532	0.349	0.417

**Table 8:** Factor correlation

factor correlation			
4 factor model	factor correlation	2 factor model	factor correlation
1-2	-0.599	1-2	0.769
1-3	-0.276		
1-4	-0.266		
2-3	0.211		
2-4	0.144		
X^2^ (*df*=566)	0.433	X^2^ (*df*=566)	0.319
null-model X^2^ (*df*= 566)	0.436	null-model X^2^ (*df*= 566)	0.398
fit index	0.466	fit index	0.401

### Subgroups differences

Correlations of C-SSRS with age and educational level were almost null and statistically non-significant (*r* = 0.23 and -0.37 respectively). A significant difference was found for gender with bi-serial point correlation (*r*= 0.59, *p* <0.021) and independent t-test (*t*(988) = -2.01, *d*= 0.66, *p* <0.025). Mantel-Haenszel (α*MH*) test showed statistical gender differences only in non-specific suicidal thoughts and controllability (*p* <0.01). A positive correlation was found between drug or alcohol use and C-SSRS (*r*= 0.59, *p* <0.015), and independent *t*-test revealed that users scored higher in suicide ideation and behavior (*t*(988)= 4.09, *d*= 0.68, *p* <0.022). Social support was negatively correlated with ideation severity (*t*(988) = -3.14, *d*= 0.64, *p* <0.036).

### Receiver Operating Characteristic (ROC) Analyses

**Figure 1:**
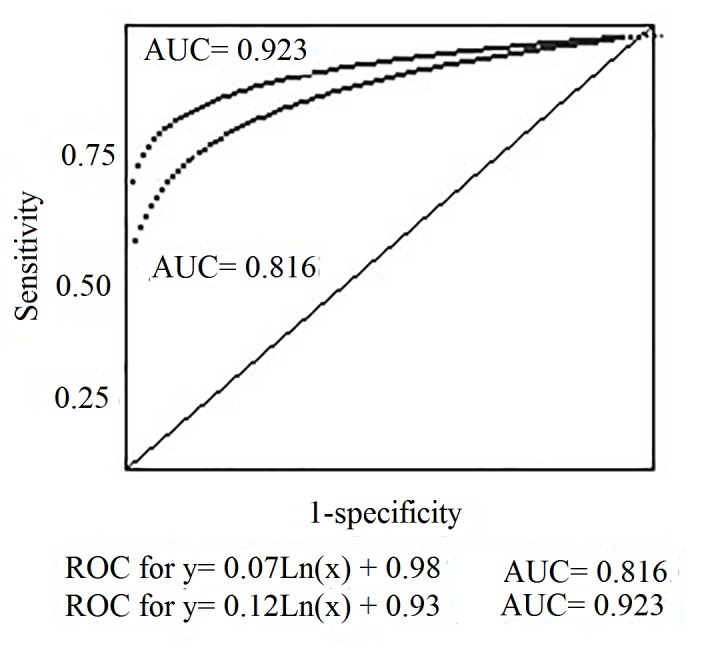
ROC values for the C-SSRS subscales

ROC analyses and cutoff scores of C-SSRS are shown in Figure 1. When ideation scale was used, a cutoff score ≥6 (out of 30) had the greatest sensitivity (94.0%) and specificity (97.9%), with adequate PPV (75.3%) and NPV (94.7%) values. Area under the Curve (AUC) was high (0.923, 95% CI= 0.843-0.965). For suicide behavior scale we obtained a cutoff score ≥4 (out of 10) with highest sensitivity (79.7%) and specificity (89.6%), with adequate PPV (78.5%) and NPV (97.1%). The AUC index (0.816; *SE*= 0.022, 95% CI= 0.722-0.917) indicated medium accuracy. 

## Discussion

In spite of high suicidal ideation rates in non-clinical high-school students, little attention has been paid to validation of self-report instruments with this population. Based on this study, the C-SSRS appears to be valid and reliable. Factor analysis supports a two-dimensional model, including suicide ideation and behavior. Cronbach's alpha estimates for C-SSRS indicate a high internal consistency. Convergent validity with other suicidality scales suggests they assess a common construct. However non-redundant, non-overlapping outcomes could be observed with PHQ-9 or SWLS, were measures like sleep, appetite disturbance or pleasurable activities do not correlate with C-SSRS subscales. EFA revealed a factor structure slightly different from that proposed by the authors ^25^. CFA with fit estimates not biased by sample size (e.g., RMSEA), or model complexity (e.g., NNFI) showed that a two-factor model had best fit indices compared to a 4 factor model and was the most appropriate solution. Although C-SSRS assess suicide risk based on current thoughts about suicide and history of self-harm or threats to commit suicide, only actual thoughts could be examined with the present study. In a previous study ^49^ it was found that individuals who have plans to commit suicide or have made up a decision to act on them were at more risk than those who only had suicide wishes or ideation. In that sense, the C-SSRS evaluates the full spectrum of suicidality, endorsing not only some vague or ill-defined thoughts about suicide, but also more clear-cut planning and preparation. Moreover, since most of suicide victims die on first attempt the detection of current ideation about suicide remains an important issue when considering suicide prevention. Using the traditional approach, we detected a floor effect range between 10 and 12% for the different C-SSRS subscales, and a ceiling effect below 3% for the intensity subscale, eliciting appropriate scaling properties. This means that the instrument is able to detect even subtle suicide thoughts in participants otherwise unsuspected of having them, as usually happens in general population. No ceiling effects were found. Results of this study suggest that the C-SSRS is useful in differentiating between non-suicide and suicide risk adolescents. Non-significant associations of C-SSRS scores with age and educative levels may enable its application to other populations besides university students. Setting a risk point for suicide is somehow difficult due to its low frequency in the general population but the results of the criterion validity analysis lend further support for establishing such a cut-off point. Taken together, the evidence indicates that the C-SSRS is a useful instrument to assess suicidality risk in adolescents. The primary benefit of this questionnaire is the flexible approach of the client, easiness for question management and the possibility it offers to use the total and scale scores in a variety of ways. Rather than asking participants to complete separate measures of ideation and behaviors, all the necessary data can be generated with this one questionnaire. Some limitations must be considered. The non-clinical composition of sample reduced the level of suicidality, as no psychiatrically diagnosed patients with high suicidality risk were assessed. The sample was also limited in terms of level of instruction diversity, so there is a risk that questions become less appropriate when assessing suicidality in minority group members, such as those with lower educational degree. Resembling previous research, gender differences in scale scores were found in this study; albeit minimal. In order to spread these encouraging results, more studies should be necessary with more diverse population. As test-retest reliability process was not employed, we can´t ensure that present results remain stable over time. Additionally, the C-SSRS would require to be evaluated with psychiatric patients, besides normal adolescents employed in the present study. Further research with the C-SSRS is required to determine if different cutoff score must be established for populations other than non-clinical adolescents, for example elder adults or individuals currently being treated for suicidality. 

## Conclusion

We presented data supporting the reliability and validity of the C-SSRS in a nonclinical sample of university Spanish-speaking students. It appears that this questionnaire is a useful instrument, both as a research and a clinical tool, combining feasible administration and flexible scoring system. This questionnaire yields data on four distinct aspects of suicidality, being in consequence more parsimonious than separate administration of a bunch of questionnaires to participants. 
