# Supplementary material for: Psychometric validation of the Columbia-Suicide Severity rating scale in Spanish-speaking adolescents
Source: Colomb Med (Cali). 2017 Dec 30;48(4):174–82. doi: 10.25100/cm.v43i4.2294 (PMC5896724; doi:10.25100/cm.v43i4.2294)
Supplement: Related texts

# Research article: Validación psicométrica de la escala de Columbia de severidad de suicidio en adolescentes hispano-hablantes

## Introduction 

El suicidio es una de los problemas más importantes de salud pública y todavía no ha sido resuelto. Las tasas de suicidio han aumentado en todo el mundo con un estimado de más de 800.000 muertes y casi diez intentos de suicidio por cada deceso. Según la Organización Mundial de la salud, cada 40 segundos una persona se suicida siendo la segunda causa de muerte entre los 15-29 años ^1^^,^^2^. Las altas tasas de suicidio son una preocupación generalizada, sobre todo en Europa Occidental ^(^^3^ y Central ^4^, los EE.UU. ^5^, los países asiáticos como Rusia y las ex Repúblicas Socialistas Soviéticas ^6^, Corea del Sur ^6^, China ^7^, India ^8^, Sri Lanka ^9^; y los países de Latinoamérica como Cuba ^10^, Uruguay ^11^; Ecuador ^12^, Bolivia ^13^, Brasil ^14^, Argentina ^15^ y Colombia ^16^. El riesgo de suicidio aumenta en personas impulsivas o con trastornos mentales, aquellos que enfrentan situaciones de estrés o que tienen fácil acceso a medios letales tales como envenenamiento, ahorcamiento y armas de fuego ^17^. Además de lo anterior, el suicidio es tres veces más común en hombres comparados con las mujeres ^18^. El suicidio es un riesgo importante en los adolescentes. En un reciente estudio ecológico sobre la mortalidad por suicidio entre 19 países de América desde el 2001 al 2008, la tasa media de suicidio para personas entre 10 y 24 años fue de 5,7/100,000 (varones: 7.7/100,000, mujeres: 2.4/100,000) y la prevalencia de ideación, planes y tentativas de suicidio estimada durante toda la vida para los jóvenes entre 13 a 18 años fueron de 12.1%, 4.0% y 4.1%, respectivamente. El miedo, la angustia y el abuso de sustancias fueron los predictores más significativos ^19^. Las tasas de suicidio adolescente están aumentando en Argentina (7.9/100,000), y disminuyendo en Canadá y Colombia ^20^. La ideación suicida es un factor de riesgo para los intentos y los suicidios consumados y lo mismo es cierto para los intentos de auto lesiones no suicidas ^21^. A pesar de la importancia de la evaluación precisa del riesgo de suicidio entre los jóvenes, muy pocos instrumentos han demostrado ser lo suficientemente confiables para este propósito debido a la baja validez o probabilidad predictiva ^22^. La Escala de Evaluación de Severidad de Riesgo Suicida de Columbia (C-SSRS) está disponible de forma gratuita en www.cssrs.columbia.edu y ha sido ampliamente utilizada para la evaluación de riesgo suicida por varias agencias tales como el Centro para soluciones integrales de salud de la Administración de Salud Mental de los EE.UU., el Instituto Nacional de Salud Mental de los EE.UU., la Administración de Drogas y Alimentos de los EE.UU., la Biblioteca Nacional de Medicina de los EE.UU., la Organización Mundial de la Salud (OMS), la guía de mejores prácticas de la Asociación Médica Americana (AMA), el Ministerio de Salud de Canadá, la Asociación Coreana de prevención del suicidio, el Instituto Nacional de Salud Mental y Neurología del Japón y el Ministerio de la Defensa de Israel ^23^. La C-SSRS fue desarrollada como parte del estudio Tratamiento del Suicidio Adolescente (TASA), que evalúa el riesgo de suicidio en diferentes escenarios clínicos y de ensayo tanto en instituciones psiquiátricas de hospitalización, como en centros de atención primaria, ambulatoria y de urgencia. Esta escala evalúa el momento de mayor gravedad e intensidad de la ideación suicida a lo largo de toda la vida, y el tipo y letalidad de la conducta suicida. Estos elementos seleccionados son fuertes predictores del riesgo de suicidio, incluyendo la actividad preparatoria ^24^. Esta escala ha sido traducida a 103 idiomas, incluyendo el español. Las propiedades psicométricas de la C-SSRS se evaluaron en tres estudios doble ciego multicéntricos realizados sobre adolescentes, los cuales mostraron alta confiabilidad interna (α = 0.73-0.95) y buena validez convergente (r = 0.80) con instrumentos de evaluación de suicidio bien reconocidos ^25^. En comparación con el Cuestionario de Historial de Suicidio de Columbia, la C-SSRS tenía mayor especificidad y sensibilidad para identificar correctamente los intentos de suicidio abortados e interrumpidos a lo largo de toda la vida. Una versión automatizada de la C-SSRS con uso de tecnología interactiva de respuesta de voz (e-C-SSRSTM) demostró una alta capacidad predictiva y moderada tasa de sensibilidad y especificidad ^26^. En otro estudio ^27^ la e-C-SSRSTM mostró mayor sensibilidad y especificidad que el ítem 9 del Cuestionario de Salud del Paciente (PHQ-9) para la predicción del suicidio (95.0% y 95.0% vs 92.0% y 81.0% respectivamente). Se encontró una gran confiabilidad entre evaluadores de la C-SSRS para discriminar comportamientos suicidas y no suicidas, y para detectar cinco categorías diferentes de comportamientos suicidas (Kappa = 0.90 y 0,88, respectivamente) en una investigación prospectiva de seguimiento de adolescentes con conductas delictivas hasta la edad adulta temprana. La sub-escala de la C-SSRS de intensidad de ideación suicida predijo tanto la frecuencia de consulta al servicio de urgencias como los intentos de suicidio futuros ^27^. Durante un estudio exploratorio ^28^ para examinar validez concurrente de la Escala de Pensamientos Suicidas-Plus, la escala de detección de suicidalidad de Sheehan y la C-SSRS para detectar auto-agresiones e ideación y comportamiento suicidas, las tres escalas mostraron un acuerdo aceptable en la detección de ideación pasiva y activa; suicidio consumado; acciones preparatorias; y comportamiento perjudicial auto-dirigido, pero solamente la C-SSRS fue capaz de detectar categorías combinadas o el intento abortado o interrumpido. La versión traducida al Español de la C-SSRS no se ha validado para población de habla hispana de América Latina, y teniendo en cuenta que las escalas deben validarse respetando los valores culturales y lingüísticas de la población para la cual están destinadas, Este estudio fue diseñado para evaluar las propiedades psicométricas de la C-SSRS en una muestra de estudiantes Universitarios de América Latina. Los objetivos específicos fueron: a) evaluar la consistencia interna, validez de criterio y la estimación de confiabilidad de la C-SSRS mediante análisis factorial confirmatorio, b) establecer puntuaciones de corte para la escala mediante análisis de las características operativas del receptor (ROC) y c) examen de sensibilidad, especificidad, valores predictivos positivos y negativos para C-SSRS.

## Materiales y Métodos

### Participantes

Los sujetos fueron reclutados al azar en una universidad pública de Argentina. El tamaño de la muestra se calculó según la fórmula siguiente:

$$n=\frac{t2Xp\left(1-p\right)}{m2}$$ (1)

en la cual n es el tamaño de muestra necesario, t es el nivel de confianza al 99%, p es la prevalencia estimada de suicidio en la zona, y m es el margen de error de 5%. Se incluyeron estudiantes que asistían a cursos de la Universidad entre el 1 de marzo de 2015 y el 1 de mayo de 2016, con edades comprendidas entre 18 y 35 años, y se realizaron análisis psicométrico exploratorio y confirmatorio en dos grupos seleccionados al azar con 782 y 834 participantes respectivamente (N = 1.616). Los registros de 78 encuestados de ambos grupos fueron descartados debido a datos incompletos. La muestra final incluyó a un primer grupo con 737 participantes, 442 mujeres (60%) y 295 hombres (40%); y a un segundo grupo con 801 participantes, 489 mujeres (61%) y 312 hombres (39%). La edad media de los participantes fue de 25.6 ±7.3. No se encontraron diferencias estadísticas entre ambos grupos en edad (hombres 23.13± 5.43 vs mujeres 24.15 ±7.01; t(988) = 1.87, p = 0.80, ns). El consentimiento informado se obtuvo para todos los encuestados. El protocolo de investigación fue aprobado por el Comité de Ética de la Facultad. Las entrevistas cara a cara fueron realizadas por investigadores capacitados en la administración de la C-SSRS, y los participantes completaron el conjunto de pruebas en pequeños grupos de 15 a 20 durante el mismo día de clases regulares.

### Instrumentos

*La version breve de la Encuesta de Salud de 12 ítems* ^29^ es una medida de auto reporte de 12 ítems de la calidad de vida relacionada con la salud y evalúa 8 dimensiones: funcionamiento físico, limitación de rol físico, dolor corporal, salud general, vitalidad, funcionamiento social, limitación de rol emocional, y salud mental, y proveen puntajes para salud física y emocional (los mayores puntajes tienen peor calidad de vida)

*La version breve del Cuestionario de Apoyo Social* ^30^ que asigna puntajes para la satisfacción con el apoyo recibido que van desde 1 (muy satisfecho) hasta 6 (muy insatisfecho); el puntaje de corte ≥2 indica insatisfacción con el apoyo social.

*Test de Identificación de Trastorno en el uso de Alcohol (AUDIT* ^31^ ha sido validado para su uso en la comunidad; se empleó un puntaje de corte de ≥8 para identificar el consumo riesgoso; 

*Uso de drogas* las preguntas indagaban si el participante alguna vez había empleado o consumido drogas prescriptas para trastornos médicos, y se clasificaban las repuestas como si (1) o no (0).

*Datos demográficos* Incluyeron género declarado por el estudiante y nivel socio-económico (SES), el cual se codificó como una variable binaria con 1 (encima) y 0 (debajo) del salario mínimo nacional.

*La escala de Ideación Suicida (SSI)* ^32^ es una escala semi-estructurada administrada por el clínico, y consiste en 19 ítems que evalúan los deseos suicidas activos y pasivos y los planes específicos. Cada ítem se puntúa en una escala de Likert de 3 puntos con un nivel de corte ≥ 6. Los mayores puntajes se asocian con mayor severidad de la ideación suicida.

*El Inventario de Depresión de Beck—II (BDI-II)* ^33^^)^ es un instrumento de auto-reporte de 21 ítems para evaluar la severidad de la depresión. Los items se puntúan de 0 a 3 y los resultados van desde 0-9 (mínimo), 10-16 (leve), 17-29 (moderado), y 30-63 (severo). El BDI-II tiene una consistencia interna adecuada (0.93). 

*El Cuestionario de Comportamientos Suicidas Revisado (SBQ-R)* ^34^ evalúa además de la ideación de suicidio, la frecuencia, amenazas, intentos y la probabilidad de suicidio. Tiene un valor alfa de Cronbach de 0.87 y un punto de corte ≥7 con una sensibilidad entre 83.0% y 92.5% y especificidad entre el 90.6% - 96.0%; en pacientes psiquiátricos adolescentes hospitalizados una puntuación de 8 se asocia con una sensibilidad del 80% y una especificidad del 91%. 

*Escala de Desesperanza de Beck (BHS)* ^35^ es un instrumento de auto-reporte con respuestas verdadero o falso compuesto por 21 ítems que miden la desesperanza acerca de los futuros eventos. Tiene buenas propiedades psicométricas. La severidad de la desesperanza se mide como: 0-3 mínima, 4-8 leve, 9-14 moderada, y 15-20 severa. La desesperanza se asocia con ideación e intentos suicidas en adolescentes y adultos jóvenes ^36^

*Escala de Satisfacción con la Vida (SWLS)* ^37^^)^ es un cuestionario de auto-reporte de 5 ítems, que evalúa la satisfacción con la vida. Los items se puntúan con una escala de Likert de 7 puntos, y tiene un coeficiente alfa de Cronbach de 0.87 y adecuada validez temporal (*r*_tt_ = 0.54) a los 4 años. 

*La Escala de Afectos Positivos y Negativos (PANAS)* ^38^ es un instrumento de auto-reporte de 20 ítems para valorar los afectos positivos (PANAS-PA; 10 ítems) y negativos (PANAS-NA; 10 ítems). Los ítems se valoran con una escala de tipo Likert de 5 puntos, y tiene una adecuada confiabilidad y validez para las escalas PANAS-PA y PANAS-NA (α de Cronbach = 0.88 y 0.84 respectivamente).

*PHQ-9*^39^ es un instrumento auto administrado para detectar depresión mayor y se enfoca en los últimos 14 días, pregunta sobre la frecuencia en que el paciente se ha sentido preocupado con algo o alguien. Los ítems se puntúan con una escala de 0-3, e incluyen poco placer o interés, sentirse deprimido, trastornos del sueño, fatiga, trastornos del apetito, sentimientos de fracaso o culpa, problemas de concentración, retardo psicomotor o agitación, e ideas suicidas. Los puntajes de 5, 10, 15, y 20 corresponden a niveles de depresión leve, moderado, moderadamente severo, y severo.

*Escala de Trastornos Afectivos y Esquizofrenia para jóvenes en edad escolar -Actual y a lo largo de toda la vida (KSADS-PL)* ^40^ es una entrevista diagnóstica semi-estructurada. El comportamiento suicida se determina con 4 ítems: ítem 1 pensamientos suicidas, ítem 2 y 3 severidad y letalidad del intento suicida, y el ítem 4 de comportamiento auto lesivo sin intencionalidad suicida. Se considera como un instrumento confiable para los adolescentes con respecto al riesgo suicida, y tiene una confiabilidad ponderada kappa de 0.87.

La Escala de Evaluación de Severidad de Riesgo de Suicidio de Columbia (C-SSRS) fue desarrollada por investigadores de la Universidad de Columbia, Pennsylvania y Pittsburgh, para evaluar el comportamiento y la ideación suicida, habiendo sido traducida a múltiples idiomas incluyendo el español. Se compone de cuatro categorías: gravedad e intensidad de la ideación suicida, y severidad y letalidad del comportamiento suicida. El Instrumento utiliza como escala temporal la evaluación a lo largo de la vida y el momento de mayor gravedad. La versión de la escala que toma en cuenta el período de vida más reciente registra las tendencias suicidas pasadas y presentes en el último mes, y la versión sobre la última visita evalúa el cambio en las tendencias suicidas desde la última consulta del paciente. La versión de la escala para tamizado se utiliza en entornos de atención de urgencias y en centros de llamadas de crisis para los usuarios de servicios de salud en general que no tienen trastornos mentales diagnosticados. En el presente estudio las categorías del C-SSRS fueron adaptadas para facilitar las definiciones y mejorar el informe de los resultados (Tabla 1). La sub-escala de Ideación suicida tiene 6 elementos mutuamente excluyentes incluidos en una escala ordinal (puntuación total: 0-30): severidad (0-5), frecuencia (0-5), duración (0-5), motivos (0-5), medidas preventivas (0-5) y controlabilidad (0-5). La sub-escala de comportamiento suicida tiene 2 ítems mutuamente excluyentes puntuados en una escala ordinal (puntaje total: 0-10): intensidad (0-5) y letalidad (0-5). En el caso de que cualquier participante manifestara una ideación suicida active con un plan o intencionalidad manifiesta de llevar a cabo el acto, se lo instaba a acordar una cita y evaluación con un equipo de salud mental o se lo conducía a una sala de emergencias para asegurar una correcta atención del evento. 

**Tabla 1:** Categorías y sub-ítems de la C-SSRS

Categoria	Item
Categoría I: Severidad de intención suicida	1: deseo de estar muerto
	2 pensamientos activos suicidas no específicos
	3: ideación suicida activa con cualquier método (sin plan) sin intención de llevarlo a cabo
	4: : ideación suicida activa con alguna intención de actuar, sin plan específico
	5: ideación suicida active con plan específico e intención de llevarlo a cabo
II: Intensidad de la ideación suicida	6: frecuencia (desde 1: < una vez semanal a 5: varias veces en el día)
	7: duración (desde1: fugaces hasta 5: más de 8 horas/persistentes o continuos)
	8: controlabilidad (desde 1: fácilmente controlables hasta 5: incapaz de controlarlos)
	9: disuasivos (desde 1: detienen el suicidio hasta 5: definitivamente no detienen el suicidio)
	10: razones (desde1: atención, venganza a 5: terminar el dolor (no puedo tolerar el dolor)
III: comportamiento suicida	11: actos o comportamientos preparatorios
	12: intento abortado
	13: intento interrumpido
	14: intent actual (no fatal)
	15: comportamiento auto-lesivo sin intención suicida
IV: Letalidad	16: sin daño físico o muy leve
	17: daño físico menor
	18: daño físico moderado; necesita atención médica.
	19: daño físico moderado/severo; debe hospitalizarse y posibles cuidados intensivos.
	20: daño físico severo; requiere internación con cuidados intensivos
	21: muerte

### Procedimiento

Los participantes completaron los cuestionarios socio-demográficos incluyendo evaluación de calidad de vida, consumo de medicamentos o alcohol y apoyo social. Después de administrar los instrumentos clínicos para evaluar estado de ánimo depresivo, riesgo de suicidio y calidad de vida, se aplicó finalmente la C-SSRS. La muestra se dividió en dos subgrupos de riesgo suicida y sin riesgo o de control para evaluar la capacidad discriminante de la C-SSRS. Los criterios de inclusión para el subgrupo de riesgo de suicidio incluyeron: 1) puntuación total del SBQ-R de ≥7 ^41^

2) puntaje total del BDI-II ≥10 (síntomas depresivos leves a severos) ^42^

3) un puntaje en la K-SADSPL de 2 en el ítem 1 (ideación suicida leve) y un puntaje de 2 en cualquiera de los ítems 2-4 (actos suicidas de intensidad leve) independientemente de la ideación ^43^, y

4) un puntaje en el PHQ-9 ≥8 ^44^

Utilizando estos criterios, los 138 participantes (9% de la muestra), con 82 mujeres y 56 hombres fueron asignados para el subgrupo de riesgo de suicidio, en tanto que los restantes 1,400 participantes (91%) fueron para el subgrupo de control. No se encontraron diferencias entre ambos grupos en cuanto a la edad (M = 23.8 ±5.38) y el género.

Análisis estadístico 

La comparación entre variables cuantitativas fueron reportadas usando la media y desviación estándar (±SD); de lo contrario se utilizó la prueba t de Student. Las variables categóricas fueron analizadas por prueba de χ2 o exacta de Fisher. El Grupo 1 se utilizó para realizar un análisis factorial exploratorio. La consistencia interna se determinó con el coeficiente alfa de Cronbach. Efecto techo y suelo se calcularon para no superar el límite del 15% de los participantes. Se evaluó la correlación de elementos con coeficiente de rango de Spearman bivariada y para el tamaño de efecto se calculó la d de Cohen. La validez de constructo de la C-SSRS se evaluó con análisis de componentes principales (PCA) y rotación Promax. Fueron utilizadas las pruebas de Kaiser-Meyer-Olkin y de esfericidad de Bartlett para determinar la adecuación de datos para el procesamiento factorial. Los factores escalares fueron extraídos mediante la prueba de correlación parcial mínima de Velicer y pruebas de sedimentación. Para el análisis factorial confirmatorio (CFA) se empeló el grupo 2 (N = 801), utilizando el programa EQS ^43^. Los datos fueron tratados como variables continuas usando una correlación de orden cero entre factores (modelo predeterminado) y una estructura de 1, 2 y 4 factores (modelos restrictivos). Las cargas y correlaciones factoriales en el primer modelo (predeterminado) se establecen en 0 y se liberan para que varíen con distintos grados de libertad de ajuste, mientras que en los modelos restrictivos las cargas se limitan para ajustarse a 1, 2 y 4 factores. Se empleó la corrección de Satorra-Bentler para X^2^ para ajustarse a la no normalidad de los datos. El criterio de bondad de ajuste fue fijado en 0,90 para el Indice incremental de ajuste (IFI) ^45^, el indice de ajuste comparative (CFI), el indice de bondad de ajuste (GFI), el indice de bondad de ajuste restrictivo (AGFI) y el índice de ajuste normativo (NFI); 0.80 para la media del error cuadrático de aproximación (RMSEA) ^46^; 0.7 para el índice de ajuste no normativo (NNFI) y el índice de ajuste parsimonioso (PNFI) y un rango 2:1 o 3:1 para la discrepancia minima de Chi-cuadrado dividido por los grados de libertad (CMINDF). La validez concurrente se examinó con las escalas SSI, BDI-II, SBQ-R, BHS, SWLS, PANAS, y KSADS-PL usando una Correlación de Pearson**.** La validez de criterio se determinó aplicando test *t* y un MANOVA de una vía entre los subgrupos de riesgo suicida y control. La contribución relativa de al riesgo suicida de los factores sociodemográficos y psiquiátricos, junto con la sub-escalas de la C-SSRS se determinó mediante un análisis de regresión múltiple stepwise ^47^. El análisis ROC se realizó para evaluar las propiedades discriminantes de la C-SSRS, y el punto de corte óptimo se determinó mediante el índice de Youden entre sensibilidad y especificidad ^48^. Para todos los test se aceptó un nivel de significación de 0.01 %. Los análisis se realizaron con el paquete estadístico SPSS 15.0 (SPSS, Chicago, IL, USA) 

## Resultados 

En la Tabla 2 se describen los datos sociodemográficos y las puntuaciones en la C-SSRS. La media de la C-SSRS para los dos grupos 1 y 2 (N = 1.538) fue de 0.76 y 1.21 respectivamente. Las mujeres tuvieron puntuaciones más altas que los varones (1.29 ±0.45 vs ±0.65 1.11, t = 0.765, *p* <0.09, respectivamente) aunque esta diferencia no fue estadísticamente significativa.

**Tabla 2:** Datos demográficos de participantes (incluye grupos 1 and 2)

Variable	Hombre (n = 607)	Mujer (n = 931)	*p* <0.01
Edad (Rango)	18-35	18-35	ns
Edad (media ± SD)	23.13±5.43	24.15±7.01	ns
Educación (Rango)	13-18	13-20	ns
Educación (media ± SD)	15.80 ± 2.2	17.1±3.5	0.31
AUDIT	0.59±0.11	0.45±0.19	0.02
Versión breve 12 ítem Calidad Vida	3.34±1.37	3.46±1.16	0.51
Versión breve Cuestionario Apoyo Social	1.24±0.12	1.32±0.30	0.34
C-SSRS total (media ± SD)	1.14±0.35	1.38±0.22	0.05
Puntaje severidad suicidio (media ± SD)	0.12±0.13	0.18±0.12	0.14
Puntaje comportamiento suicida (media± SD)	0.22±0.07	0.20±0.12	0.01
Puntaje intensidad suicidio (media ± SD)	1.21±0.16	2.18±0.17	0.04
Puntaje letalidad suicidio (media ± SD)	1.18±0.16	1.09±0.12	0.01
PHQ-9	5.21±1.11	5.98±1.99	0.56
SSI	2.21±1.17	2.12±1.19	0.39
BDI-II	7.19±2.11	8.65±2.40	0.62
SBQ-R	3.19±1.09	4.22±1.43	0.82
BHS	3.17±1.18	3.00±1.64	0.23
SWLS	20.99±6.10	25.01±7.31	0.43
PANAS-PA	28.11±12.10	33.46±10.19	0.29
PANAS-NA	17.19±8.12	18.93±8.55	0.29
KSADS-PL item 1	1.06±0.22	1.04±0.19	0.29
KSADS-PL item 2	1.08±0.21	1.10±0.32	0.22
KSADS-PL item 3	1.14±0.09	1.10±0.12	0.33

SSI: escala Ideación Suicida; BDI-II: Inventario depresión Beck; SBQ-R: Cuestionario comportamiento suicida-Revisado; escala desesperanza de Beck (BHS); SWLS: escala satisfacción con la vida; PANAS-PA: escala afectos positivos; PANAS-NA: escala afectos negativos; KSADS-PL: escala de trastornos afectivos y esquizofrenia para jóvenes en edad escolar-actual y a lo largo de toda la vida

### Consistencia Interna 

La sub-escala de ideación suicida de la C-SSRS tuvo un índice de fiabilidad de mitades de Guttmann de.91 y un alfa de Cronbach de 0.87, 0.89 y 0.93 para toda la muestra y los grupos de riesgo de suicidio y control respectivamente, con una buena consistencia interna. Las sub-escalas de intensidad y gravedad de ideación suicida y severidad y letalidad de comportamiento suicida tuvieron un alfa de Cronbach de 0.73, 0.89 y 0.91 respectivamente. Las puntuaciones más bajas de los efectos techo y suelo fueron encontradas en el 11.8%, 10.5%, 10.1% y 11.3% de los casos para las sub-escalas de intensidad y severidad de ideación suicida y gravedad y letalidad de comportamiento suicida, respectivamente. Las puntuaciones más altas fueron para la sub-escala de intensidad (2.4% de los ítems). La media de correlación entre ítems para la C-SSRS fue 0.64 (mínimo 0.47, máximo 0.72). La correlación corregida entre puntuación total e ítems de la C-SSRS se presenta en la Tabla 3, con valores que van desde 0.47 para el ítem 6 y 0.74 para el ítem 5 (*p* < 0.001).

**Tabla 3:** Consistencia interna y estadístico Mantel-Haenszel de la C-SSRS (αMH)

Item	M	SD	r_**tot**_	α	α_**MH**_
Severidad intención suicida					
1. Deseo de estar muerto	0.15	0.01	0.62	0.926	0.68
2. Pensamientos suicidas no específicos	0.12	0.02	0.49	0.929	1.04*
3. Ideación suicida activa con cualquier método (sin plan) sin intención de llevar a cabo	0.13	0.04	0.54	0.926	1.30
4. Ideación suicida activa con intención de llevar a cabo, sin plan específico	0.11	0.03	0.56	0.926	1.44
5. Ideación suicida activa con plan específico e intención de llevar a cabo	0.14	0.01	0.74	0.926	0.89
Intensidad de ideación suicida					
6. Frecuencia (desde 1: <una vez semanal a 5: muchas veces por día)	1.35	0.73	0.47	0.925	2.41
7. Duración (desde 1: fluctuantes a 5: más de 8 horas/persistente o continuo)	1.56	0.79	0.67	0.925	1.31
8. Controlabilidad (desde 1: fácil control a 5: incapaz de control)	2.43	0.98	0.55	0.923	0.99*
9. Disuasivos (desde 1: detiene suicidio a 5: no detiene suicidio)	2.05	0.87	0.54	0.924	0.89
10. Razones (desde1: atención, venganza a 5: detener dolor	1.26	0.65	0.72	0.925	1.14
Comportamiento suicida					
11. Acto o comportamiento preparatorio	0.22	0.17	0.65	0.925	1.11
12. Intento abortado	0.17	0.05	0.63	0.925	0.84
13. Intento interrumpido	0.24	0.10	0.48	0.924	1.34
14. Intento actual (no fatal)	0.15	0.09	0.50	0.926	1.48
15. Comportamiento auto-lesivo sin intención suicida	0.21	0.19	0.61	0.925	1.33
16. Sin daño físico	0.12	0.07	0.53	0.928	1.18
Letalidad suicidio					
17. Daño físico menor	1.80	0.39	0.54	0.925	1.95
18. Daño moderado; requiere atención médica	0.84	0.46	0.61	0.928	1.53
19. Daño severo; requiere hospitalización	1.01	0.32	0.64	0.932	1.49
20. Daño severo; requiere hospitalización con cuidados intensivos	1.03	0.27	0.57	0.930	1.37

*Estadísticamente significativo con *p* <0.01

### Validez factorial 

Se llevó a cabo un análisis factorial exploratorio de eje principal con rotación PROMAX para la matriz de correlación de la C-SSRS, ya que tanto el test Kaiser-Meyer-Olkin (KMO = 0.92) y el test de esfericidad de Bartlett (χ2 = 2,740.4, *p* < 0.001) indicaron la adecuación muestral para el procesamiento factorial. El test de Velicer de correlación mínima parcial y el test de sedimentación arrojaron una solución de 2 factores. Todos los ítems tenían una carga factorial ≥0.40 (Tabla 4)

**Tabla 4:** Cargas factorials de la C-SSRS

**Item**	Patron de Matriz		Matriz de estructura		Comunalidades	
	**1** ^**er**^ **Factor**	**2** ^**do**^ **Factor**	**1** ^**er**^ **Factor**	**2** ^**d0**^ **Factor**	**inicial**	**extracción**
1. Deseo de estar muerto	**0.83**	0.19	**0.85**	0.25	1.00	0.80
2. Ideación activa no específica	**0.82**	0.14	**0.82**	0.18	1.00	0.79
3. Ideación activa sin plan ni intención	**0.80**	0.17	**0.78**	0.21	1.00	0.78
4. Ideación activa con intención sin plan específico	**0.79**	0.29	**0.77**	0.25	1.00	0.76
5. Ideación activa plan específico e intención	**0.76**	0.23	**0.74**	0.26	1.00	0.73
6. Frecuencia (1: <una vez/semana - 5: muchas veces por día)	**0.75**	0.13	**0.73**	0.20	1.00	0.72
7. Duración (1: Fugaces -5: >8 horas/persistentes o continuas)	**0.74**	0.18	**0.72**	0.28	1.00	0.71
8. Controlabilidad (1: fácil control -5: incapaz de control)	**0.73**	0.17	**0.70**	0.27	1.00	0.69
9. Disuasivos (1: detiene suicidio -5: no detiene)	**0.71**	0.11	**0.68**	0.20	1.00	0.67
10. Razones (1: atención, venganza -5: terminar dolor)	**0.70**	0.10	**0.65**	0.19	1.00	0.64
11. Actos preparatorios	−0.23	**0.86**	0.18	**0.82**	1.00	0.79
12. Intento abortado	−0.27	**0.82**	0.09	**0.81**	1.00	0.76
13. Intento interrumpido	−0.30	**0.80**	0.23	**0.78**	1.00	0.72
14. Intento actual (no fatal)	−0.19	**0.78**	0.35	**0.77**	1.00	0.69
15. Auto-lesión sin intento suicida	0.36	**0.77**	0.08	**0.76**	1.00	0.68
16. Sin daño físico	0.18	**0.76**	0.19	**0.74**	1.00	0.68
17. Daño físico menor	0.14	**0.74**	0.15	**0.73**	1.00	0.71
18. Daño moderado; requiere atención médica	0.28	**0.69**	0.20	**0.70**	1.00	0.70
19. Daño severo; requiere hospitalización	0.15	**0.68**	0.13	**0.63**	1.00	0.69
20. daño severo; requiere cuidados intensivos	−0.04	**0.67**	-0.21	**0.68**	1.00	0.65

*Nota* factores de carga significativos en negrita.

Los dos factores oblicuos tenían valores eigen de 6.97 (IC 95%: 5.11, 7.75) y 4.91 (IC 95%: 3.76, 4.61) respectivamente. El primer factor tenía 10 ítems (1 a 10) consistente con el concepto de ideación suicida. El segundo factor incluía 10 ítems (11 a 20) reflejando el comportamiento suicida. Ambos factores estaban correlacionados positive y moderadamente entre sí (r= 0.67). 

### Validez concurrente 

El análisis de varianza multivariado (MANOVA) de una vía con las puntuaciones ponderadas de las dos sub-escalas de la C-SSRS como variables dependientes y el género como variable independiente demostró diferencias de género significativas, ya que las mujeres tenían puntuaciones más altas en ideación suicida con un puntaje en Hotelling T2 = 0,03; F(4, 630) y un valor en test exacto = 4.67, *p* <0.01, d = 0.79. En tanto que las mujeres tenían menores puntajes en comportamiento suicida (F (4, 630) = 3.86, *p* <0.05, d= 0.82), lo cual representa un hallazgo usual en la población general. La C-SSRS tuvo una relación positiva con SSI (r= 0.71, d= 0.87, *p* <0.001), BDI-II (r= 0.77, d= 0.84, *p* <0.002), SBQ-R (r= 0.47, d= 0.40, *p* <0.099), BHS (r= 0.59, d= 0.47, *p* <0.017), PANAS-NA (r= 0.70, d= 0.88, *p* <0.002), ítem 1 de la KSADS PL (r= 0.87 d= 0.88, *p* <0.001) y correlación negativa con la SWLS (r= -0.58 d= 0.12, *p* <0.099) y PANAS PA (r= -0.63 d= 0.20, *p* <0.045) (Tabla 5).

**Tabla 5:** Correlaciones concurrentes de la C-SSRS con otras escalas

	Ideación suicida			Comportamiento		
	r	d	*p*	r	d	*p*
Items PHQ-9						
Poco interés o placer	0.45	0.75	0.045	0.32	0.46	0.076
Depresión	0.55	0.81	0.071	0.41	0.45	0.071
Trastorno sueño	0.32	0.52	0.043	0.49	0.50	0.055
Fatiga	0.41	0.60	0.042	0.32	0.56	0.063
Trastorno apetito	0.33	0.45	0.077	0.33	0.48	0.067
Sentimientos de culpa	0.51	0.61	0.049	0.43	0.62	0.073
Dificultad de concentración	0.22	0.60	0.071	0.39	0.70	0.075
Retardo psicomotor o agitación	0.31	0.51	0.049	0.48	0.69	0.083
Ideas suicidas	0.89	0.90	0.003	0.52	0.80	0.009
SSI	0.71	0.87	0.001	0.43	0.32	0.087
Deseo suicida activo	0.70	0.85	0.002	0.40	0.23	0.032
Plan específico	0.71	0.82	0.001	0.41	0.31	0.063
Deseo pasivo suicida	0.56	0.43	0.087	0.21	0.23	0.066
BDI-II	0.77	0.84	0.004	0.49	0.22	0.032
SBQ-R	0.47	0.40	0.099	0.79	0.81	0.009
BHS	0.59	0.47	0.017	0.50	0.54	0.018
SWLS	-0.58	0.12	0.099	0.11	0.32	0.034
PANAS PA	-0.63	0.20	0.045	0.13	0.32	0.078
PANAS NA	0.70	0.88	0.002	0.66	0.79	0.004
Items K-SADS-PL						
Item 1 ideas suicidas	0.87	0.88	0.001	0.31	0.43	0.012
Item 2 severidad intento	0.31	0.44	0.032	0.78	0.87	0.005
Item 3 letalidad intento	0.12	0.53	0.049	0.50	0.77	0.015
Item 4 auto-destructivo	0.27	0.22	0.067	0.32	0.41	0.070

Coeficiente de correlación bivariado de Spearman; SSI: Scale for Suicide Ideation, BDI-II: Beck Depression Inventory-II, SBQ-R: Suicidal Behaviors Questionnaire-Revised; BHS: Beck Hopelessness Scale, SWLS: Satisfaction with Life Scale, PANAS: Positive and Negative Affect Schedule, KSADS-PL: Schedule for Affective Disorders and Schizophrenia for School; d Cohen: <0.3 (efecto pequeño), 0.5 (efecto medio), >0.8 (efecto grande)

### Validez de criterio

El test *t* entre los subgrupos de riesgo suicida y control mostró significativas diferencias (1.98 ±0.67 vs. 0.31 ±0.12, respectivamente; *t*(760)= -10.21, *p* <0.001, d= 4.56). El análisis de regresión múltiple stepwise permitió evaluar la validez discriminante de las subescalas de la C-SSRS como variables independientes para predecir el riesgo de suicidio (Tabla 6). Los factores sociodemográficos se introdujeron en l primera instancia, seguidos por las escalas clínicas y subescalas de la C-SSRS. Las escalas de ideación y comportamiento suicidas contribuyeron significativamente para la predicción de riesgo de suicidio (coeficiente= 0.58, t= 4.34, *p* <0.001; OR= 6.21, IC 95%= 5.62-7.87 y coeficiente = 0.61, t= 5.12, *p* <0.001, OR= 6.22, IC 95%= 6.00-7.91, respectivamente). La R^2^ ajustada aumentó (R^2^= 0.87, F(9,880)= 1,564.31, d= 6.76 *p* <0.001) después que las subescalas de la C-SSRS se introdujeron en la ecuación. 

**Tabla 6:** Análisis de regresión logística de subescalas C-SSRS para predecir suicidio

Variable	β	SE	Wald	OR (IC 95%)	χ^**2**^	*R* ^***2***^
Constante	-0.01	-0.11	0.14	0.91 (0.86-1.00)	0.36	0.12
*Paso 1*						
genero (0=femenino)	0.01	-0.12	0.25	1.03 (0.89-1.18)	0.65	0.34
Educación	0.07	0.31	0.45	1.06 (0.59-1.28	0.55	0.43
SES	0.21	0.21	0.39	1.12 (0.87-1.21	0.31	0.54
*Paso 2*						
AUDIT	0.06	0.22	0.36	2.21 (1.69-2.52	0.43	0.49
IQOL 12-item	0.35	0.48	0.17	1.98 (0.77-1.88)	032	0.31
SSQ	0.37	0.20	0.15	2.11 (1.32-2.68)	0.21	0.48
*Paso 3*						
C-SSRS subescalas	1.99	0.65	5.98	6.87 (4.87-8.43)	6.87***	0.87
Ideas suicidas	1.67	0.78	6.31	6.21 (5.62-7.87)	7.12***	0.76
Conducta suicida	1.87	0.55	7.31	6.22 (6.00-7.91)	8.32**	0.82

** p <0.01; *** p < 0.001

### Analisis factorial confirmatorio (AFC)

El resultado del AFC con los modelos predeterminado y restringidos de 1, 2 y 4 factores, junto con los puntajes de las correlaciones factoriales se muestran en las Tablas 7 y 8. El modelo de 2 factores produjo el mejor índice de ajuste.

**Tabla 7:** Estadísticos de bondad de ajuste

	X^**2**^	*df*	significancia	CMINDF	IFI	RMSEA	CFI	GFI	AGFI	NFI	NNFI	PNFI
Modelo default (2 factores)	0.455	566	0.23	4.091	0.817	0.142	0.821	0.623	0.531	0.487	0.518	0.491
Modelos restrictictivo												
1 factor	0.497	566	0.65	1.098	0.946	0.067	0.947	0.910	0.901	0.899	0.893	0.890
2 factores	0.288	566	0.01	0.872	0.976	0.038	0.980	0.998	0.994	0.997	0.992	0.995
4 factores	0.528	566	0.09	5.673	0.821	0.099	0.812	0.341	0.369	0.532	0.349	0.417

**Table 8:** Factor correlation

factor correlación			
4 factor model	factor correlation	2 factor model	factor correlation
1-2	-0.599	1-2	0.769
1-3	-0.276		
1-4	-0.266		
2-3	0.211		
2-4	0.144		
X^2^ (*df*=566)	0.433	X^2^ (*df*=566)	0.319
null-model X^2^ (*df*= 566)	0.436	null-model X^2^ (*df*= 566)	0.398
fit index	0.466	fit index	0.401

### Diferencias entre subgrupos 

Las correlaciones de la C-SSRS con la edad y niveles educacionales fueron mínimas y no significativas (*r*= 0.23 and -0.37 respectivamente). Se encontró una diferencia significativa para género analizadas con punto de correlación bi-serial (*r*= 0.59, *p* <0.021) y test-t independiente (*t*(988)= -2.01, d= 0.66, *p* <0.025). El test de Mantel-Haenszel (α*MH*) mostró diferencias significativas entre géneros solo para ideación suicida no específica y para el factor de controlabilidad (*p* <0.01). Se encontró una correlación positiva entre el uso de drogas y alcohol y la C-SSRS (*r*= 0.59, *p* <0.015), y un test-t para muestras independientes reveló que los usuarios tenían mayores puntajes en las subescalas de ideación y comportamiento suicidas (*t*(988)= 4.09, d= 0.68, *p* <0.022). El apoyo social correlacionó negativamente con la severidad de ideación suicida (*t*(988) = -3.14, *d*= 0.64, *p* <0.036).

### Análisis ROC 

Este análisis junto con los puntos de corte de la C-SSRS se muestra en la Figura 1. Para la escala de ideación suicida un punto de corte ≥6 (de un total de 30 puntos) tuvo la mayor sensibilidad (94.0%) y especificidad (97.9%), con valores adecuados de PPV (75.3%) NPV (94.7%). El área bajo la curva (AUC) fue alta (0.923, IC 95%= 0.843-0.965). Para la escala de comportamiento suicida se obtuvo un puto de corte ≥4 (de un total de 10 puntos) con la mayor sensibilidad (79.7%) y especificidad (89.6%), con valores adecuados de PPV (78.5%) NPV (97.1%). El índice de AUC (0.816; *SE*= 0.022, IC 95%= 0.722-0.917) indicó una precisión alta. 

**Figura 1:** ROC para las subescalas de C-SSRSs

## Discusión

A pesar de las altas tasas de suicidio entre estudiantes sin patología clínica, se ha prestado poca atención a la validación de escalas de auto-reporte para esta población. Basándonos en el presente estudio, la C-SSRS resulta ser un instrumento válido y confiable. El análisis factorial apoya una solución de dos factores, incluyendo la ideación y el comportamiento suicidas. Las estimaciones alfa de Cronbach para la C-SSRS señalan una alta consistencia interna, en tanto que la validez convergente con tras escalas de riesgo de suicido sugiere que evalúan un constructo común a todas. Sin embargo se observaron resultados de correlación no redundantes ni colineares con las escalas PHQ-9 o SWLS, donde otras medidas tales como sueño, trastorno del apetito o actividades placenteras no correlacionan con las subescalas de la C-SSRS. El AFE mostró una estructura factorial discretamente diferente de la propuesta por los autores ^25^. El AFC con estimaciones de ajuste no sesgadas por el tamaño de la muestra (ej., RMSEA), o la complejidad del modelo (ej., NNFI) mostraron que el modelo de dos factores tenía mejores índices de ajuste comparado con los modelos de cuatro factores y representaba una solución más apropiada. Aunque la C-SSRS evalúa el riesgo de suicidio basándose en los pensamientos actuales sobre suicidio y el historial de comportamientos o amenazas suicidas y/o de auto-lesiones sin intención de morir, en el presente estudio solo se pudieron explorar los pensamientos y los comportamientos actuales. En un estudio anterior ^49^ se encontró que los individuos que planean cometer un suicidio o han tomado la decisión de llevarlo a cabo tenían mayor riesgo que aquellos que solo tenían ideación suicida. En este sentido la C-SSRS evalúa el espectro completo de suicidalidad tomando en consideración no solamente pensamientos o ideas vagas o mal definidas sobre suicidio sino también los planes y preparativos más concretos. Más aun, dado que la mayoría de las víctimas de suicidio mueren en el primer intento, la detección de ideación actual sobre suicidio sigue siendo un tema importante desde el punto de vista de la prevención. Usando el enfoque tradicional, detectamos un rango de efecto techo entre 10 y 12% para las diferentes subescalas de la C-SSRS, y un rango de efecto techo por debajo de 3% para la sub-escala de intensidad, demostrando propiedades escalares apropiadas. Esto significa que el instrumento es apto para detectar aun sutiles ideas de suicidio en participantes que de otro modo hubieran pasado inadvertidos como ocurre usualmente en la población general. No se encontró efecto techo significativo. Los resultados de este estudio sugieren que la C-SSRS es útil para diferenciar entre adolescentes con y sin riesgo de suicidio. Las asociaciones no significativas de la C-SSRS con los niveles educativos y la edad de los participantes permiten aplicar el instrumento en diferentes contextos sociales además de estudiantes universitarios. Establecer un puto de riesgo para suicidio es un poco complicado debido a la baja frecuencia de ocurrencia en la población general pero los resultados del análisis de validez de criterio constituyen un robusto apoyo para establecer un punto de corte. En conjunto la evidencia indica que la C-SSRS es un instrumento útil para evaluar el riesgo de suicidio en adolescentes. El beneficio primario de este cuestionario es que permite un abordaje flexible del paciente, facilidad para completar y puntuar las respuestas en una gran variedad de formatos. En lugar de obligar a los participantes a completar distintas medidas de ideación y comportamiento suicida, la escala ofrece la posibilidad de completar todas las medidas con un suelo cuestionario. Algunas limitaciones a ser consideradas son la composición de la muestra integrada por personas sin diagnóstico clínico lo cual puede haber reducido el nivel de riesgo de suicido, en tanto no hubo pacientes diagnosticados con trastornos mentales que podrían haber exhibido mayores niveles de suicidalidad. La muestra también fue reducida en términos de diversidad de instrucción, por lo cual podría haber un sesgo de que las preguntas fuesen menos apropiadas si se emplearan para evaluar la suicidalidad en grupos minoritarios como aquéllos con menor nivel educativo. De la misma manera que en estudios anteriores, se observaron diferencias de género en los puntajes de la escala; aunque estas diferencias fueron mininas. Para obtener una generalización de estos resultados alentadores se necesitan llevar a cabo estufidos con poblaciones más diversas. Como no se empleó una prueba de confiabilidad test-retest no se puede asegurar que el presente estudio fuera estable a través del tiempo. Además la validación de la C-SSRS debería ser replicada con pacientes con trastornos mentales además de adolescentes normales tal como se realizó en el presente estudio. Se requiere ulterior investigación con la C-SSRS para determinar si se deben establecer otros puntos de corte para poblaciones distintas de adolescentes sin patología clínica, como es el caso de adultos mayores o pacientes en tratamiento por trastornos mentales con ideación suicida. 

## Conclusión

Se presentan datos que apoyan la confiabilidad y validez de la C-SSRS en una muestra no clínica de estudiantes universitarios hispano-hablantes. El cuestionario demostró ser un instrumento útil tanto para la investigación como para la evaluación clínica, combinando la flexibilidad de su administración con puntajes de amplio rango para cada una de las medidas. El cuestionario ofrece datos sobre cuatro aspectos diferentes de la suicidalidad siendo en consecuencia mas practico que la administración separada de varios instrumentos adicionales.
